# *XPG* rs873601 G>A contributes to uterine leiomyoma susceptibility in a Southern Chinese population

**DOI:** 10.1042/BSR20181116

**Published:** 2018-09-14

**Authors:** Zhi-Qin Liu, Guan-Ge Chen, Ru-Liang Sun, Chao Chen, Mei-Yin Lu, Lan-Fang Guan, Xiao-Ling Chi, You-Qiang Jian, Xiu Zhu, Rui-Qi Liu, Bo-Yu Cai, Fang-Fang Chen, Bin Liu

**Affiliations:** 1 Department of Obstetrics and Gynecology, Bao’an Maternal and Child Health Hospital, Jinan University, Shenzhen 518102, Guangdong, China; 2 Department of Obstetrics and Gynecology, The Second Affiliated Hospital and Yuying Children’s Hospital of Wenzhou Medical University, Wenzhou 325027, Zhejiang, China; 3 Department of Pathology, Bao’an Maternal and Child Health Hospital, to Jinan University, Shenzhen 518102, Guangdong, China; 4 Department of Biobank, Bao’an Maternal and Child Health Hospital, Jinan University, Shenzhen 518102, Guangdong, China

**Keywords:** DNA repair, genetic susceptibility, polymorphism, uterine leiomyoma, XPG

## Abstract

*XPG* gene contributes to DNA repair defects and genomic instability, which may lead to the initiation of uterine leiomyoma. We hypothesized that genetic variants of *XPG* gene may alter the carriers’ susceptibility to leiomyoma. The association between five potential functional single nucleotide polymorphisms (SNPs), i.e. rs2094258 C>T, rs751402 C>T, rs2296147 T>C, rs1047768 T>C, rs873601 G>A, and uterine leiomyoma risk in Chinese, was investigated in this case–control study, which included 398 incident leiomyoma cases and 733 controls. We found that rs873601 was significantly associated with tumor risk in a recessive genetic model after being adjusting for age and menopause. When compared with rs873601 GG/GA genotypes, the AA genotype had an increased leiomyoma risk (adjusted OR = 1.59, 95% CI = 1.16–2.18, *P*=0.004; Bonferroni adjusted *P*=0.040). Furthermore, stratified analysis revealed that the association between the rs873601 AA genotype and leiomyoma risk was more evident among subjects younger than 40 years old (adjusted OR = 1.58, 95% CI = 1.06–2.35, *P*=0.023) and patients who had more than three myomas (adjusted OR = 2.05, 95% CI = 1.24–3.41, *P*=0.006). Yet, no significant association between the other four polymorphisms and leiomyoma risk was observed. To sum up, the present study reported on the association between *XPG* gene polymorphisms and myoma risk. The observed data indicated that SNP rs873601 G>A contributes to uterine leiomyoma susceptibility in a Southern Chinese population.

## Introduction

Uterine leiomyoma, also known as myomata or fibroids, is the most common pelvic tumor in women [1]. Increased serum oxidative stress, which induces DNA lesions, has been associated with this type of benign tumor [2]. Likewise, environmental tumorigenic agents can also damage DNA, while different DNA repair mechanisms have been reported to alleviate such DNA damages [3]. Therefore, the polymorphisms of some DNA repair genes have been reported associated with leiomyoma risk [4–6].

Xeroderma pigmentosum group G, also known as XPG, RAD2 or ERCC5, is a 1,186-amino acid structure-specific endonucleases, which belongs to the nucleotide excision repair (NER) pathway, one of five known major DNA repair mechanisms [7]. The endonucleases XPG is important in maintaining genomic stability. XPG is a DNA damage recognition protein that binds and cleaves damaged DNA, which is followed by the excision of a 24- to 32-bp segment containing the bulky adduct at the 3’ and 5’ ends of the damaged site; finally, the resultant gap is filled by DNA synthesis and ligation [8]. In addition, XPG is involved in the RNA transcription through interaction with other transcription activator complexes, such as TFIIH [9], RNA polymerase II [10] and Gadd45a [11], which eventually influences mutagenesis and cell death. This protein is encoded by *XPG* gene, which is located on human chromosome 13q22-q33, spans 69kb length, contains 15 exons, and is highly polymorphic [12]. A serious of reports have revealed the association between the *XPG* gene polymorphisms and tumor risk, including colorectal cancer [13], gastric cancer [14–16], lung cancer [17], head and neck cancer [18], and neuroblastoma [19]. Nevertheless, no associations between *XPG* gene and leiomyoma risk have been reported so far. We hypothesized that genetic variants of *XPC* gene may modulate the carriers’ susceptibility to uterine leiomyoma.

Therefore, we conducted the current case–control study in a Southern Chinese population to understand the associations between the potential functional polymorphisms of *XPG* gene and the risk of uterine leiomyoma.

## Materials and methods

### Study population

Three hundred and ninety-eight patients with incidentally histologically confirmed leiomyoma and 733 healthy controls without uterine tumor (or other diseases), verified by ultrasonic examination, were enrolled at Bao’an Maternal and Child Health Hospital, Jinan University between January 2015 and February 2018. The respond rate of patiens and controls were 98.8% and 85.9%, respectively. All the research subjects were unrelated ethnic Han Chinese population from Southern China.

First, demographic characteristics (age and menopause), and tumor characteristics, including, numbers, sites, and diameters were obtained from all patients. Next, 2 ml of venous blood sample was collected from each subject after interview and signing the consent form.

The present study was approved by the Ethics Committee of the Bao’an Maternal and Child Health Hospital, Jinan University (IRB No: LLSC2018-02-01).

### SNPs selection and genotyping

The potentially functional single nucleotide polymorphisms (SNPs) were selected by using the NCBI dbSNP database and SNPinfo (http://snpinfo.niehs.nih.gov/snpinfo/snpfunc.htm). The applied criteria were described earlier [13,15], briefly as following: (1) the minor allele frequency reported in HapMap was more than 5% for Chinese Han subjects; (2) SNPs were located in the 5’-flanking region, exon, 5’-untranslated region (5’-UTR) and 3’-UTR, which might affect transcription activity and the microRNA-binding site activity and (3) SNPs were in low linkage disequilibrium with each other (*R*^2^ < 0.8). The widely reported SNP rs17655 G>C was excluded because of its linkage disequilibrium (LD) with rs873601 G>A (*R*^2^ = 0.91). As a result, five potential functional SNPs (rs2094258 C>T, rs751402 C>T, rs2296147 T>C, rs1047768 T>C, and rs873601 G>A) were included in the present study.

Genomic DNA was extracted from blood samples using the Qiagen Blood DNA Mini Kit (Qiagen Inc., Valencia, CA, U.S.A.) according to the manufacturer’s instructions of the manufacturer. As described previously [20], we performed genotyping of above SNPs was performed by the Taqman real-time polymerase chain reaction method using a 7900 Sequence Detection System (Thermo Fisher Scientific, Waltham, MA, U.S.A.). To achieve more reliable genotyping results, four duplicated positive controls and four negative controls without DNA template were loaded in each of 384-well plates. Genotyping was repeated on 10% of the samples randomly selected from the subjects, and the results were 100% concordant.

### Statistical analysis

Statistical analyses were performed as described earlier [21]. Briefly, we compared the differences between cases and controls regarding demographic characteristics, such as age and menopause, by using Chi-square test and Student’s *t* test; then we tested whether the genotype frequency distribution of each polymorphism in controls was in Hardy–Weinberg equilibrium through Goodness-of-fit χ^2^ test. Odds ratios (ORs) and 95% confidence intervals (CIs) were calculated to estimate the associations between each SNP and gastric cancer risk, using univariate and multivariate logistic regression models. Bonferroni correction was used to correct for multiple comparisons of SNPs, that is, the Bonferroni adjusted *P* value = (*P* value of tested SNP) × *k*!/(2!(*k −* 2)!), where *k* was the number of total SNPs. Further stratification analysis by age, menopause, and tumor characteristics (numbers, sites, and diameters) was also performed. All statistical analysis was performed using SPSS software (version 18.0; SAS Institute Inc., Chicago, IL, U.S.A.). A two-sided statistical significance level of 0.05 was chosen.

## Results

### Subject’s characteristics

The clinical and demographic characteristics of the study population, including 398 leiomyoma cases and 733 healthy controls, was described as Supplementary Table S1. Compared with controls, the cases were more likely to be younger (for subjects <40 years, 68.8% vs. 39.4%, *P*<0.001) and reproductive females (98.2% vs. 67.3%, *P*<0.001). Among the leiomyoma cases, nearly 40% (156 cases) had more than two myomas, and they were more commonly located in intramural (66.8%, 266 cases) and subserous (20.9%, 83 cases) region.

**Table 1:** Association between *XPG* gene polymorphisms and uterine fibroid risk

Genotypes	Cases, *n* (%)	Controls, *n* (%)	HWE P^*^	OR (95% CI)	*P*	AOR (95% CI)	*P*^†^
*rs2094258 C>T*							
CC	167 (42.0)	330 (45.0)	0.813	1.00		1.00	
CT	178 (44.7)	328 (44.7)		1.07 (0.83–1.39)	0.599	1.09 (0.82–1.43)	0.562
TT	53 (13.3)	75 (10.3)		1.40 (0.94–2.08)	0.100	1.48 (0.95–2.29)	0.081
Dominant	231 (58.0)	403 (55.0)		1.13 (0.89–1.45)	0.322	1.16 (0.89–1.51)	0.285
Recessive	345 (86.7)	658 (89.8)		1.35 (0.93–1.96)	0.119	1.42 (0.94–2.14)	0.100
*rs751402 C>T*							
CC	150 (37.6)	271 (37.0)	0.553	1.00		1.00	
CT	194 (48.4)	358 (48.8)		0.98 (0.75–1.28)	0.876	0.95 (0.71–1.26)	0.718
TT	54 (13.6)	104 (14.2)		0.94 (0.64–1.38)	0.745	0.89 (0.59–1.34)	0.573
Dominant	248 (62.3)	462 (63.0)		0.97 (0.75–1.25)	0.812	0.94 (0.71–1.23)	0.628
Recessive	344 (86.4)	629 (85.8)		0.95 (0.67–1.35)	0.774	0.92 (0.63–1.34)	0.648
*rs2296147 T>C*							
TT	257 (64.6)	443 (60.4)	0.171	1.00		1.00	
CT	121 (30.4)	260 (35.5)		0.80 (0.62–1.03)	0.089	0.80 (0.60–1.07)	0.128
CC	20 (5.0)	30 (4.1)		1.15 (0.48–2.74)	0.754	1.20 (0.81–2.97)	0.679
Dominant	141 (35.4)	290 (39.6)		0.84 (0.66–1.07)	0.152	0.86 (0.66–1.14)	0.294
Recessive	378 (95.0)	703 (95.9)		1.24 (0.60–-2.57)	0.564	1.32 (0.78–3.19)	0.579
*rs1047768 T>C*							
TT	209 (52.5)	367 (50.0)	0.338	1.00		1.00	
CT	154 (38.7)	311 (42.4)		0.87 (0.67–1.13)	0.287	0.86 (0.65–1.13)	0.282
CC	35 (8.8)	55 (7.5)		1.12 (0.71–1.76)	0.634	1.10 (0.67–1.81)	0.703
Dominant	189 (47.5)	366 (49.9)		0.91 (0.71–1.16)	0.432	0.90 (0.69–1.17)	0.410
Recessive	363 (91.2)	678 (92.5)		1.19 (0.77–1.85)	0.444	1.18 (0.73–1.91)	0.505
*rs873601 G>A*							
GG	108 (27.1)	201 (27.4)	0.305	1.00		1.00	
GA	183 (46.0)	381 (52.0)		0.89 (0.67–1.20)	0.453	0.83 (0.61–1.14)	0.251
AA	107 (26.9)	151 (20.6)		1.32 (0.94–1.86)	0.111	1.41 (0.97–2.06)	0.071
Dominant	290 (72.9)	532 (72.6)		1.02 (0.77–1.33)	0.918	0.98 (0.73–1.32)	0.900
Recessive	291 (73.1)	582 (79.4)		**1.42 (1.07–1.89)**	**0.016**	**1.59 (1.16–2.18)**	**0.004**

Notes: *Goodness-of-fit χ^2^ test; ^†^adjusted for age, and menopause

Abbreviations: AOR, adjusted OR; CI, confidence interval; HWE, Hardy–Weinberg equilibrium; OR, odds ratio.

Statistically significant associations are indicated by bold text.

### Associations between *XPG* gene polymorphisms and leiomyoma risk

Table 1 summarized the genotype distributions of the selected *XPG* gene polymorphisms in all subjects. The genotype frequency distributions of all SNPs in the control subjects were in agreement with Hardy–Weinberg equilibrium (all *P*>0.05, Table 1).

Next, we examined the association between the above morphisms and myoma risk. Variables including age and menopause were adjusted for in the subsequent multivariate logistic regression analyses (Table 1). The logistic regression analysis showed that polymorphism rs873601 G>A was significant associated with tumor risk in a recessive genetic model after adjusting for age and menopause. When compared with rs873601 GG+GA genotypes, the rs873601 AA variant genotype had an increased leiomyoma risk (adjusted OR = 1.59, 95% CI = 1.16–2.18, *P*=0.004; Bonferroni adjusted *P*=0.040). While no associations between other four polymorphisms (rs2094258 C>T, rs751402 C>T, rs2296147 T>C, and rs1047768 T>C) and tumor risk were observed in either of the three genetic models.

### Stratification analysis

We further investigated the potential association between the most important polymorphism rs873601 G>A of *XPG* gene and the leiomyoma risk in the stratified study by age, menopause, and tumor characteristics (numbers, sites, and diameters) (Table 2). The rs873601 AA variant genotype was found to be associated with a significantly increased risk of uterine leiomyoma among individuals younger than 40 (adjusted OR = 1.58, 95% CI = 1.06–2.35, *P*=0.023), when GG+GA genotypes served as the reference. Similarly, when compared with the reference genotypes, carriers of rs873601AA genotype had a significantly increased risk of leiomyoma among reproductive females (adjusted OR = 1.65, 95% CI = 1.20–2.28, *P*=0.002), cases with one myoma (adjusted OR = 1.55, 95% CI = 1.08–2.24, *P*=0.019) or more than three myomas (adjusted OR = 2.05, 95% CI = 1.24–3.41, *P*=0.006), subjects with myomas located in subserous (adjusted OR = 2.60, 95% CI = 1.56–4.32, *P*<0.001), and myomas’ diameter less than 5 mm (adjusted OR = 1.96, 95% CI = 1.31–2.94, *P*=0.001).

**Table 2:** Stratification analysis for association between *XPG* rs873601 G>A genotypes and uterine fibroid risk

Genotypes	rs873601 G>A (cases/controls)		OR (95% CI)	*P*	AOR (95% CI)	*P* ^†^
	GG+GA	AA				
Age, years						
<40	200/234	74/55	**1.58 (1.06–2.34)**	**0.025**	**1.58 (1.06–2.35)**	**0.023**
≥40	91/348	33/96	1.32 (0.83**–**2.08)	0.242	1.65 (0.98**–**2.77)	0.066
Menopause						
No	285/403	106/90	**1.67 (1.21–2.29)**	**0.002**	**1.65 (1.20–2.28)**	**0.002**
Yes	6/179	1/61	0.49 (0.06**–**4.14)	0.512	0.59 (0.07**–**5.12)	0.630
No. of myoma						
1	178/582	64/151	1.39 (0.99**–**1.94)	0.058	**1.55 (1.08–2.24)**	**0.019**
2	54/582	16/151	1.14 (0.64**–**2.05)	0.657	1.31 (0.72**–**2.39)	0.380
≥3	59/582	27/151	**1.76 (1.08–2.88)**	**0.023**	**2.05 (1.24–3.41)**	**0.006**
Site of myoma*						
Intramural	204/582	62/151	1.17 (0.84**–**1.64)	0.356	1.32 (0.92**–**1.90)	0.128
Subserous	52/582	31/151	**2.30 (1.42–3.71)**	**0.001**	**2.60 (1.56–4.32)**	**<0.001**
Other types	35/582	14/151	1.54 (0.81**–**2.94)	0.188	1.80 (0.93**–**3.49)	0.081
Diameter, mm*						
≤5.0	110/582	50/151	**1.74 (1.19–2.54)**	**0.004**	**1.96 (1.31–2.94)**	**0.001**
>5.0	180/582	57/151	1.22 (0.86**–**1.73)	0.261	1.39 (0.95**–**2.02)	0.086

Notes: *in the biggest myoma; ^†^adjusted for age, and menopause.

Abbreviations: AOR, adjusted OR; CI, confidence interval; OR, odds ratio.

Statistically significant associations are indicated by bold text.

Because of very few subjects in the subgroups, such as menopause females, some subgroups were not significantly associated with the risk of leiomyoma risk.

## Discussion

In the present study, we found that *XPG* polymorphism rs873601 G>A was associated with an increased leiomyoma risk. In addition, this association was more evident among younger subjects and those with multiple myomas. To the best of our knowledge, this is the first study that reported on the association of *XPG* polymorphisms with uterine leiomyoma.

Some studies have investigated the role of *XPG* polymorphisms in different other tumors. In an Eastern Chinese population, rs873601A variant genotypes (GA+AA) was associated with a significantly elevated risk of gastric cancer [15]. However, the association between this SNP and gastric cancer has not been validated in a Southern Chinese population in another study [22]. Moreover, Wang et al. [23] reported that this SNP was associated with hepatocellular cancer risk by single-locus analysis only in screening stage. Besides, Hua et al. [24] reported that rs873601 A allele can also contribute to the susceptibility of colorectal cancer in a Southern Chinese population with a total of 1,901 cases and 1,976 controls. Two studies have performed comprehensive meta-analyses to evaluate the association of *XPG* polymorphism rs873601 with cancer risk: Han et al. [25] found that polymorphism rs873601 was significantly associated with overall cancer risk, using data from 12 studies, including 9,158 cases and 10,073 controls focus on rs873601; while another meta-analysis study that included data from 23 reports found that this polymorphism was related to the cancer susceptibility only in Asians [26]. The ethnic and demographic differences among studies might be partly due to the enormously different frequencies of the rs873601 A allele in different groups (0.48 in Chinese, CHB; 0.53 in Caucasian, CEU; 0.30 in Africans (YRI), according to HAPMAP database, www.hapmap.org/). In addition to tumorigenesis, polymorphism rs873601G>A has also been reported the association with poorer disease-free survival and overall survival, in Chinese patients with esophageal squamous cell cancer receiving platinum-based adjuvant chemotherapy [27]. Combined with the above reports, our results suggested that this SNP might be used as surrogate marker for tumor risk.

*XPG* gene plays the critical role in the NER pathway. Briefly, XPG cleaves the DNA strand at the 3’ side of the damaged site and stabilizes the DNA repair complex [28–30]. Thus, functional *XPG* variants may alter the DNA repair capacity of NER, thus modifying the risk of leiomyoma. Additionally, *XPG* rs873601 is a cis-regulatory SNP, which might be related to gene expression [31]. Thereby, our results on the association of leiomyoma risk and *XPG* rs873601 G>A polymorphism are biologically plausible.

Our data suggested that the risk effect of *XPG* rs873601 AA genotypes remained significant in the subgroups of younger subjects (<40 years old) and those with multiple myomas (≥3). Youngers are usually more exposed to less environmental mutagens, thus the role of genetic variants in tumor case might outweighed than enviromental factors in tumorigenesis. In addition, we also found that the association between an increased tumor risk and this SNP (rs873601 AA) was more evident in cases carrying more myomas. Because the activities of NER are associated with cell proliferation, i.e. tumor number [31], it is possible to explain the association between this SNP and an increased risk in tumor numbers.

The present study had some limitations: first, it was a hospital-based case–control study, restricted only to a Chinese Han population. However, the genotype frequencies of all studied SNP among controls well fit the Hardy–Weinberg disequilibrium law, suggesting the subjects’ selection is in random. Second, the controls were older than the cases in the present study. Nevertheless, these selection criteria might be helpful for excluding some “future” tumor case. Third, some other risk factors should be considered in later studies, such as metabolism, hormones, and environmental factors [32].

In conclusion, our data suggested that the *XPG* rs873601 G>A polymorphism was associated with an increased leiomyoma risk. Future well-designed, prospective studies with larger sample size, involving different ethnicities, are needed to confirm these findings.

## Supporting information

**Suppl. Table 1.** Clinical and demographic characteristics of uterine fibroid patients and fibroid-free controls.

## Abbreviations

CI: confidence interval
HWE: Hardy–Weinberg equilibrium
NER: nucleotide excision repair
OR: odds ratio
SNP: single nucleotide polymorphism
