# Supplementary material for: XPG rs873601 G>A contributes to uterine leiomyoma susceptibility in a Southern Chinese population
Source: Biosci Rep. 2018 Sep 14;38(5):BSR20181116. doi: 10.1042/BSR20181116 (PMC6137253; doi:10.1042/BSR20181116)
Supplement: Supplementary file 1 [file bsr20181116_Supp1.pdf]

Suppl. Table 1. Clinical and demographic characteristics of uterine fibroid patients and fibroid-free controls.

Variables	Cases,n (%)	Controls, n (%)	*P* [b]
All subjects	398(100.0)	733(100.0)	
Age, yr			
< 40	274(68.8)	289(39.4)	<0.001
≥ 40	124(31.2)	444(60.6)	
Menopause			
No	391 (98.2)	493 (67.3)	<0.001
Yes	7 (1.8)	240 (32.7)	
No. of myoma			
1	242(60.8)	-	
2	70(17.6)	-	
≥3	86(21.6)	-	
Site of myoma [a]			
Intramural	266(66.8)	-	
Subserous	83(20.9)	-	
Intraligamentary	11(2.8)	-	
Submucous	34(8.5)	-	
Cervical	4 (1.0)	-	
Diameter, mm [a]			
≤ 5.0	161(40.5)	-	
> 5.0	237(59.5)	-	

Notes:[a] the characteristics of the biggest myoma; [b] chi-square test
